# Differential Melanoma outcomes in men and women: longitudinal findings from a ten-year study^؆^

**DOI:** 10.1016/j.abd.2026.501352

**Published:** 2026-04-22

**Authors:** Natália Martins Sampaio, Flávio Wellington Martins Cruz, Sebastião Maurício de Oliveira Castro, Ana Carolina Ribeiro de Oliveira, Patrícia Rocha Martins, Fabrizio dos Santos Cardoso, Sérgio Gomes da Silva, João Pereira Duprat Neto, Alice Muglia Amancio

**Affiliations:** a Department of Research, Development and Innovation, Hospital do Câncer de Muriaé, Fundação Cristiano Varella, Muriaé, MG, Brazil; b Postgraduate Program in Medical Sciences, Instituto D’Or de Pesquisa e Ensino, Rio de Janeiro, RJ, Brazil; c Medical Course, Afya Centro Universitário Itaperuna, Itaperuna, RJ, Brazil; d Study Center, Hospital São Vicente de Paulo, Bom Jesus do Itabapoana, RJ, Brazil; e Department of Skin Cancer, A.C. Camargo Cancer Center, São Paulo, SP, Brazil; f Medical Course, Centro Universitário FAMINAS, Muriaé, MG, Brazil

Dear Editor,

Cutaneous melanoma is an aggressive neoplasm arising from melanocytes and is responsible for the majority of skin cancer-related deaths. Although it represents only 4% of skin tumors, it accounts for up to 75% of skin cancer mortality due to its high metastatic potential.^1^ Global incidence has increased steadily, with 331,647 new cases estimated in 2022, reflecting 1.7% of all cancers.^2^ While early-stage melanomas have a favorable prognosis, advanced cases with greater Breslow thickness and ulceration are associated with poor outcomes.^3^

Several risk factors influence melanoma development, including UV exposure, fair skin, and genetic predisposition.^4^ Another well-established risk factor is the number of melanocytic nevi, which is independently associated with melanoma risk.^4^ Gender differences in melanoma outcomes have been noted, with women presenting at earlier stages and having better survival rates than men.^5^, ^6^ However, these disparities remain underexplored in Latin American populations. This study aimed to investigate gender-related differences in clinical presentation and survival among melanoma patients treated at a reference center in Southeast Brazil over a ten-year period.

In this study, we retrospectively collected data on the epidemiological, clinical, and histopathological data from patients diagnosed with Cutaneous Melanoma (CM) at a cancer reference hospital in southeast Brazil. The research commenced following approval from the local Research Ethics Committee (CAAE #55961622.6.0000.5105).

Our analysis included patients diagnosed between January 2010 and December 2020. Clinical data, including age, gender, socioeconomic status, tumor location, staging, and tumor histological subtype, were extracted from electronic medical records. Information regarding patient mortality was obtained through an active search, which involved families and federal databases. Among the 317 diagnosed cases, 46 were excluded due to incomplete records or inability to contact patients or families for follow-up.

Data were analyzed using descriptive statistics, with results presented as absolute and relative frequencies (%). Quantitative variables were summarized using the median and interquartile range. Categorical variables were compared using the chi-square test or Fisher's exact test, as appropriate, with a significance level set at 5% (p < 0.05). ANOVA assessed differences between groups, followed by Tukey's post hoc. The *t*-test evaluated mean age differences between sexes. All analyses were performed using *R* software version 4.2.2 (R CORE TEAM, 2022).

We identified 317 cases of cutaneous melanoma in Southeast Brazil between 2010 and 2020, of which 46 were excluded from the analysis due to incomplete medical records. The cohort comprised 136 men and 135 women (n = 271), with a mean age of 63-years. Women were diagnosed significantly younger than men (57.4 vs. 64.5 years; p = 0.0002) (Fig. 1).

**Fig. 1:**
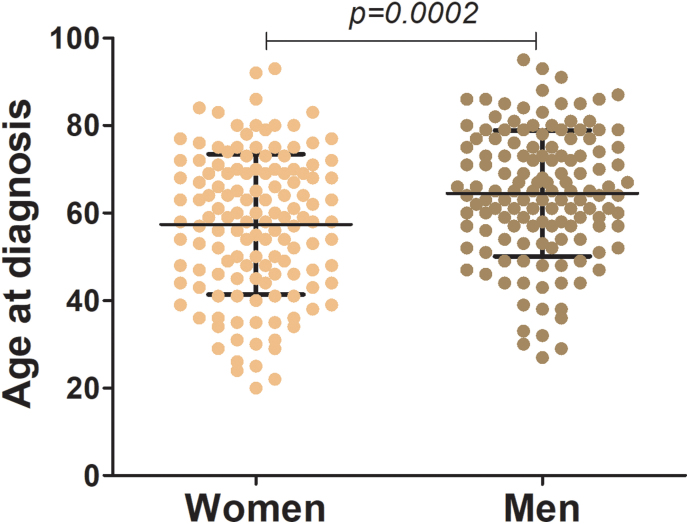
Age at diagnosis of men and women with melanoma. The figure illustrates the distribution of age at diagnosis by sex, as women were diagnosed at a younger age (57.41-years, SE = 1.38) compared to men (64.47-years, SE = 1.23; p = 0.0002).

Regarding disease staging, women were more frequently diagnosed at early stages (0–II), while men had a higher prevalence of advanced disease (III–IV) (p = 0.044). Histological subtype was unspecified in most cases (54.2%), followed by superficial spreading melanoma (21.8%), with no significant sex-based differences. The most common anatomical sites were the limbs (39.1%), followed by the trunk (29.5%) and face (19.2%), also without differences between sexes (p = 0.411).

Significant differences were observed in lifestyle habits: men reported higher rates of tobacco use (36.8% vs. 12.5%) and alcohol consumption (38.9% vs. 10.3%) compared to women (p < 0.001 for both). There were no significant differences in family history of cancer between sexes (p = 0.253). The greater Breslow thickness observed in men in our cohort reinforces this trend, suggesting both biological and behavioral components influence these outcomes.

Women were more frequently diagnosed under age 50 and at earlier stages, possibly due to greater health-seeking behavior and engagement in skin self-examinations, as described in the literature.^7^ In contrast, men reported higher rates of alcohol and tobacco use ‒ factors linked to impaired immune function and worse progression.^8^

In terms of treatment, most patients underwent surgery only (61.3%), while 15.5% received combined modalities such as surgery plus chemotherapy or immunotherapy. A smaller proportion received chemotherapy alone (4.4%), radiotherapy (1.5%), or immunotherapy alone (1.5%). No treatment ‒ usually palliative ‒ was recorded in 15.9% of patients. There were no significant differences in treatment type between men and women (p = 0.238) (Table 1).

**Table 1:** Clinical characteristics of cutaneous melanoma in men and women from Southeast Brazil.

Variable	Men (%)	Women (%)	Total (%)	p-value
Staging				0.044*
0	10 (7.4%)	18 (13.3%)	28 (10.3%)	
I	17 (12.5%)	35 (25.9%)	52 (19.2%)	
II	33 (24.3%)	26 (19.3%)	59 (21.8%)	
III	28 (20.6%)	25 (18.5%)	53 (19.6%)	
IV	30 (22.1%)	24 (17.8%)	54 (19.9%)	
Unknown*	18 (13.2%)	07 (5.2%)	25 (9.2%)	
Primary location				0.411
Limbs	54 (39.7%)	52 (38.5%)	106 (39.1%)	
Trunk	35 (25.7%)	45 (33.3%)	80 (29.5%)	
Face	30 (22.0%)	22 (16.3%)	52 (19.2%)	
Neck and sculp	10 (7.3%)	13 (9.6%)	23 (8.4%)	
Other	7 (5.1%)	3 (2.2%)	10 (3.6%)	
Alcohol use				<0.001*
Former user	21 (15.4%)	6 (4.4%)	27 (10.0%)	
Current user	32 (23.5%)	8 (5.9%)	40 (14.8%)	
No	50 (36.8%)	76 (56.3%)	126 (46.5%)	
Unknown	33 (24.3%)	45 (33.3%)	78 (28.8%)	
Tobacco use				<0.001*
Former user	34 (25.0%)	6 (4.4%)	40 (14.8%)	
Current user	16 (11.8%)	11 (8.1%)	27 (10.0%)	
No	62 (45.6%)	76 (56.3%)	138 (50.9%)	
Unknown	24 (17.6%)	42 (31.1%)	66 (24.4%)	
Family history				0.253
Yes	46 (33.8%)	43 (31.9%)	89 (32.8%)	
No	44 (32.4%)	27 (20.0%)	71 (26.2%)	
Unknown	46 (33.8%)	65 (48.1%)	111 (41.0%)	
Treatment				0.238
Surgery only	77 (28.4 %)	89 (32.8%)	166 (61.3%)	
Chemotherapy only	06 (2.2%)	06 (2.2 %)	12 (4.4%)	
Radiotherapy only	02 (0.7%)	02 (0.7%)	04 (1.5 %)	
Immunotherapy	01 (0.4%)	03 (1.1%)	04 (1.5%)	
Combination treatment	28 (10.3 %)	14 (5.2%)	42 (15.5%)	
No treatment (palliative)	22 (8.1 %)	21 (7.8%)	43 (15.9 %)	

In survival analysis (mean follow-up: 36-months), worse outcomes were observed in patients with Breslow thickness ≥ 2 mm, Clark level > III, and advanced disease stages (p < 0.001). Five-year survival was higher among women (75%) compared to men (50%) (p < 0.001) (Fig. 2). This finding is consistent with previous reports showing that men are diagnosed later and have poorer prognosis, even when controlling for tumor stage.^5^, ^6^

**Fig. 2:**
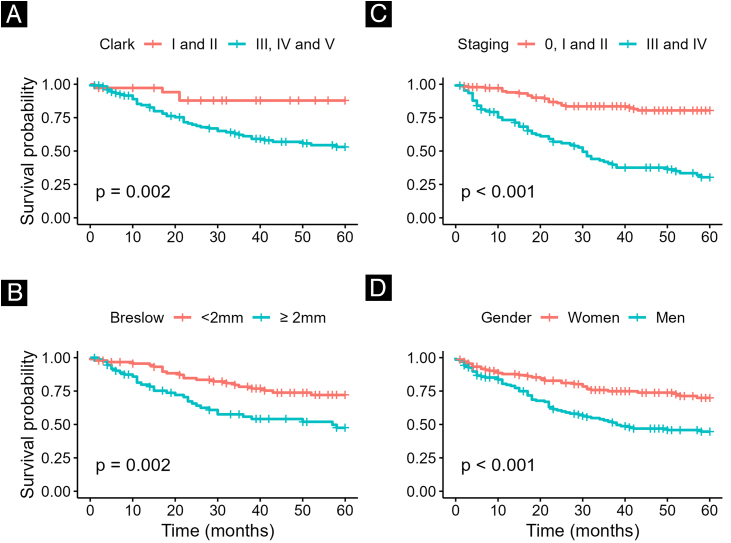
Survival analysis of patients with melanoma in Southeast Brazil according to (A) Clark level, (B) Breslow depth, (C) disease staging, and (D) gender.

Despite no significant sex differences in tumor location or treatment, survival remained lower among men, indicating that anatomical or therapeutic factors alone do not explain this disparity. As supported by previous studies, biological differences in immune response or hormonal influence may contribute to sex-specific disease behavior.^5^

Our study also reinforces the relevance of regional context. Many patients in our cohort came from rural areas, where access to dermatologic care is limited, and sun exposure is chronic, particularly among agricultural workers.^9^ These conditions may contribute to delayed diagnoses and worse prognoses, especially in men. Tailored awareness campaigns focusing on rural male populations may help close this gap.

While most patients underwent surgical treatment, advanced therapies such as immunotherapy were rarely used. During the study period (2010–2020), the use of immunotherapy was still incipient in Brazil, which explains its rare application in this cohort. Moreover, the majority of patients were treated within the Brazilian public healthcare system (SUS), where access to high-cost medications remains restricted. Although combinations like nivolumab and ipilimumab have shown survival benefits in metastatic disease,^10^ socioeconomic barriers likely limit access in public healthcare settings, underscoring the importance of prevention and early detection.

Our study has limitations. Its retrospective design may introduce bias, and the relatively short follow-up period (median 36-months) limits evaluation of long-term survival. Incomplete medical records also led to the exclusion of cases, which may also have influenced results. Our dataset did not include information on self-care behaviors, such as skin self-examination or healthcare-seeking patterns, that could help explain the differences in stage diagnosis between sexes.

Another limitation of this study is the high proportion of cases without a specified histological subtype, which may have underestimated the prevalence of lentigo maligna melanoma. Also, the grouping of early (I–II) and advanced (III–IV) stages for analysis may have masked distinct prognostic behaviors across individual stages.

Our findings demonstrate significant gender disparities in melanoma outcomes, with men presenting at older ages, with thicker tumors and more advanced disease, leading to worse survival. Taken together, our data support a multifactorial understanding of sex disparities in melanoma, shaped by biological traits, behavioral patterns, healthcare access, and socioeconomic context. Interventions must therefore combine public health efforts, clinical innovation, and equitable healthcare delivery to reduce outcome gaps and improve prognosis for all patients.

## ORCID ID

Natália Martins Sampaio: 0000-0002-4224-0286

Flávio Wellington Martins Cruz: 0009-0009-8416-8401

Sebastião Maurício de Oliveira Castro: 0000-0002-2890-8534

Ana Carolina Ribeiro de Oliveira: 0000-0001-8262-8667

Patrícia Rocha Martins: 0000-0001-6222-0293

Fabrizio dos Santos Cardoso: 0000-0002-7547-8880

Sérgio Gomes da Silva: 0000-0002-9650-809X

João Pereira Duprat Neto: 0000-0001-8968-4506

## Financial support

None declared.

## Authors' contributions

Natália Martins Sampaio: Approval of the final version of the manuscript; critical literature review; data collection, analysis and interpretation; preparation and writing of the manuscript; study conception and planning.

Flávio Wellington Martins Cruz: Approval of the final version of the manuscript; data collection, analysis and interpretation; study conception and planning.

Sebastião Maurício de Oliveira Castro: Approval of the final version of the manuscript; study conception and planning.

Ana Carolina Ribeiro de Oliveira: Approval of the final version of the manuscript; statistical analysis.

Patrícia Rocha Martins: Approval of the final version of the manuscript; critical literature review; manuscript critical review.

Fabrizio dos Santos Cardoso: Approval of the final version of the manuscript; critical literature review; manuscript critical review.

Sérgio Gomes da Silva: Approval of the final version of the manuscript; critical literature review; manuscript critical review.

João Pereira Duprat Neto: Approval of the final version of the manuscript; manuscript critical review.

Alice Muglia Amancio: Approval of the final version of the manuscript; critical literature review; data collection, analysis and interpretation; effective participation in research orientation; preparation and writing of the manuscript.

## Research data availability

The entire dataset supporting the results of this study was published in this article.

## Funding

This research received no external funding. Institutional support was provided by Fundação Cristiano Varella.

## Conflicts of interest

None declared.
